# Shifting global health governance towards the sustainable development goals

**DOI:** 10.2471/BLT.18.209668

**Published:** 2018-12-01

**Authors:** Robert Marten, Sowmya Kadandale, Anders Nordström, Richard D Smith

**Affiliations:** a London School of Hygiene and Tropical Medicine, 15-17 Tavistock Place, Kings Cross, London WC1H 9SH, England.; b Health Unit, United Nations Children’s Fund, Jakarta, Indonesia.; c Swedish Ministry of Foreign Affairs, Stockholm, Sweden.; d College of Medicine and Health, University of Exeter, Exeter, England.

The definition of global health governance is the use of formal and informal institutions, rules and processes by states, intergovernmental organizations and non-state actors to deal with health challenges that require effective cross-border collective actions.^1^ Since 2000, global health governance processes and financing allocations have largely focused on the millennium development goals (MDGs). Three out of the eight MDGs related directly to health, and the other five goals focused on critical determinants of health. The MDGs increased aid flows, particularly for health.^2^ In the early 2000s, two new funding organizations, the Global Fund to Fight AIDS, Tuberculosis and Malaria, and Gavi, the Vaccine Alliance, were created to help finance the health MDGs. Around the same time, the President’s Emergency Plan for AIDS Relief was created, with originally a five-year, 15 billion United States dollars (US$) commitment. By 2014, roughly US$ 23 billion out of a total of US$ 36 billion, or almost two-thirds of development assistance for health, were directed towards the MDGs.^3^ Efforts to reach the MDG health targets dominated global health governance and reduced policy space, and accompanying financial allocations, for the consideration of other health challenges. 

The MDG era also brought an expanding academic interest in the field of global health and global health governance. The MDGs made a critical and often overlooked contribution to the conceptualization of global health, creating a normative global health agenda that continues to be reflected in the current architecture and financing of global health governance.^5^ While building on the MDGs, the sustainable development goals (SDGs) reflect a significant enlargement for the development agenda and present an opportunity to expand the scope of global health governance. *Transforming our world: the 2030 agenda for sustainable development* positions health as a broad development issue. We argue that despite a major broadening of the focus for health there have been no reforms to global health governance. Global health governance is still mostly intended to deliver the MDGs, not the SDGs.

The SDGs, and specifically SDG 3, that is, to ensure healthy lives and promote well-being for all at all ages, require a paradigm shift in global health.^5^ This has not happened. No notable institutional, structural or financial reforms to global health governance to achieve the SDGs have taken place, and donors have not shifted their financing efforts. New health financing mechanisms are still being established to advance the unfinished MDG agenda, such as the Global Financing Facility for Every Woman Every Child (established in July 2015), which focuses on reproductive, maternal, newborn, child and adolescent health. Without any reference to the SDGs, in 2015, donors committed US$ 7.5 billion to Gavi, the Vaccine Alliance for immunization and the Global Fund’s replenishment conference in late 2016 saw donor pledges of an additional US$ 12.9 billion for HIV, tuberculosis and malaria. The World Health Organization (WHO) programme budget for 2018–2019 allocates US$ 805 million for communicable diseases in comparison to US$ 351 million for noncommunicable diseases.^6^

While efforts to meet the MDG-related health goals should obviously continue, more serious efforts and focus are now needed to meet SDG 3. With its thirteenth general programme of work,^7^ WHO has an opportunity to lead not just on achieving health security and universal health coverage, but also to define a clear strategy to promote health in sectors beyond and outside the health sector. However, this opportunity to reform and focus on SDG 3 cannot and should not be limited to WHO.^8^ Existing institutions, financial allocations and policy processes across the field of global health will need to be rethought to meet SDG 3.

With the shift from the MDGs to a more comprehensive and integrated 2030 Agenda, the SDGs will require broader financing and effective work across several sectors throughout national and global governance. Increased efforts to regulate and control risk factors for noncommunicable diseases, such as alcohol and tobacco, will also be needed. These products,^9^^,^^10^ as well as foods high in fat, salt and sugar,^11^ are increasingly consumed, but development planning, budgeting and financing rarely considers how to address these challenges and their health implications. 

While Phase One of the Global Action Plan for SDG 3 is ongoing, this new effort should enable assessing progress on all SDG 3 targets and the health-related indicators of other SDGs. The analysis for this Global Action Plan should suggest new reforms in governance, leadership and a reprioritization of financing. For global health governance, this will require approaching SDG 3 holistically rather than by individual targets, diseases or programmes and moving beyond the MDG health agenda. Existing institutions and financing instruments must be significantly reformed and if necessary, repurposed. 

## References

[1] Fidler D. The challenges of global health governance. New York: Council on Foreign Relations; 2010.

[2] Kenny C, Sumner A. More money or more development: what have the MDGs achieved? Working Paper 278. Washington, DC: Center for Global Development; 2011 Available from: https://www.files.ethz.ch/isn/142901/1425806_file_Kenny_Sumner_MDGs_FINAL.pdf [cited 2018 Jan 27].

[3] Dieleman J, Murray C, Haakenstad A. Financing global health 2014: shifts in funding as the MDG era closes. Seattle: The Institute for Health Metrics and Evaluation; 2015. http://www.healthdata.org/policy-report/financing-global-health-2014-shifts-funding-mdg-era-closes [cited 2018 Jan 27].

[4] Marten R. How states exerted power to create the millennium development goals and how this shaped the global health agenda: lessons for the sustainable development goals and the future of global health. Glob Public Health. 2018 4 26:1–16. 10.1080/17441692.2018.1468474 29697307

[5] Buse K, Hawkes S. Health in the sustainable development goals: ready for a paradigm shift? Global Health. 2015 3 21;11(1):13. 10.1186/s12992-015-0098-8 25890267 PMC4389312

[6] World Health Organization. Programme budget 2018-2019. Geneva: World Health Organization; 2017. http://apps.who.int/iris/bitstream/handle/10665/272406/WHO-PRP-17.1-eng.pdf?sequence=1&isAllowed=y [cited 2018 Nov 15].

[7] Draft thirteenth general programme of work 2019–2023. In: Seventy-First World Health Assembly, Geneva, 5 April 2018. Geneva: World Health Organization; 2018. Available from: http://apps.who.int/gb/ebwha/pdf_files/WHA71/A71_4-en.pdf?ua=1 [cited 2018 Jan 27].

[8] Smith R, Lee K. Global health governance: we need innovation not renovation. BMJ Glob Health. 2017 3 9;2(2):e000275. 10.1136/bmjgh-2016-000275 28589032 PMC5435265

[9] Report on the global tobacco epidemic, 2017: monitoring tobacco use and prevention policies. Geneva: World Health Organization; 2017. Available from: http://www.who.int/tobacco/global_report/en/ [cited 2018 Jan 28].

[10] Global status report on alcohol and health. Geneva: World Health Organization; 2014. Available from: http://apps.who.int/iris/bitstream/handle/10665/274603/9789241565639-eng.pdf?ua=1 [cited 2018 Jan 27].

[11] Monteiro CA, Levy RB, Claro RM, de Castro IR, Cannon G. Increasing consumption of ultra-processed foods and likely impact on human health: evidence from Brazil. Public Health Nutr. 2011 1;14(1):5–13. 10.1017/S1368980010003241 21211100

